# Deferoxamine Alleviates Osteoarthritis by Inhibiting Chondrocyte Ferroptosis and Activating the Nrf2 Pathway

**DOI:** 10.3389/fphar.2022.791376

**Published:** 2022-03-14

**Authors:** Zhou Guo, Jiamin Lin, Kai Sun, Jiayou Guo, Xudong Yao, Genchun Wang, Liangcai Hou, Jingting Xu, Jiachao Guo, Fengjing Guo

**Affiliations:** ^1^ Department of Orthopedics, Tongji Hospital, Tongji Medical College, Huazhong University of Science and Technology, Wuhan, China; ^2^ Michigan State University’s Broad College of Business, East Lansing, MI, United States; ^3^ Department of Orthopedic Surgery, the Second Affiliated Hospital of Chongqing Medical University, Chongqing, China; ^4^ Department of Pediatric Surgery, Tongji Hospital, Tongji Medical College, Huazhong University of Science and Technology, Wuhan, China

**Keywords:** osteoarthritis, chondrocytes, ferroptosis, deferoxamine, Nrf2

## Abstract

**Objective:** Osteoarthritis (OA) is a common disease with a complex pathology including mechanical load, inflammation, and metabolic factors. Chondrocyte ferroptosis contributes to OA progression. Because iron deposition is a major pathological event in ferroptosis, deferoxamine (DFO), an effective iron chelator, has been used to inhibit ferroptosis in various degenerative disease models. Nevertheless, its OA treatment efficacy remains unknown. We aimed to determine whether DFO alleviates chondrocyte ferroptosis and its effect on OA and to explore its possible mechanism.

**Methods:** Interleukin-1β (IL-1β) was used to simulate inflammation, and chondrocyte ferroptosis was induced by erastin, a classic ferroptosis inducer. A surgical destabilized medial meniscus mouse model was also applied to simulate OA *in vivo*, and erastin was injected into the articular cavity to induce mouse knee chondrocyte ferroptosis. We determined the effects of DFO on ferroptosis and injury-related events: chondrocyte inflammation, extracellular matrix degradation, oxidative stress, and articular cartilage degradation.

**Results:** IL-1β increased the levels of ROS, lipid ROS, and the lipid peroxidation end product malondialdehyde (MDA) and altered ferroptosis-related protein expression in chondrocytes. Moreover, ferrostatin-1 (Fer-1), a classic ferroptosis inhibitor, rescued the IL-1β–induced decrease in collagen type II (collagen II) expression and increase in matrix metalloproteinase 13 (MMP13) expression. Erastin promoted MMP13 expression in chondrocytes but inhibited collagen II expression. DFO alleviated IL-1β– and erastin-induced cytotoxicity in chondrocytes, abrogated ROS and lipid ROS accumulation and the increase in MDA, improved OA-like changes in chondrocytes, and promoted nuclear factor E2–related factor 2 (Nrf2) antioxidant system activation. Finally, intra-articular injection of DFO enhanced collagen II expression in OA model mice, inhibited erastin-induced articular chondrocyte death, and delayed articular cartilage degradation and OA progression.

**Conclusion:** Our research confirms that ferroptosis occurs in chondrocytes under inflammatory conditions, and inhibition of chondrocyte ferroptosis can alleviate chondrocyte destruction. Erastin-induced chondrocyte ferroptosis can stimulate increased MMP13 expression and decreased collagen II expression in chondrocytes. DFO can suppress chondrocyte ferroptosis and promote activation of the Nrf2 antioxidant system, which is essential for protecting chondrocytes. In addition, ferroptosis inhibition by DFO injection into the articular cavity may be a new OA treatment.

## Introduction

As a fairly common joint disease, osteoarthritis (OA) exhibits high disability and incidence rates among elderly individuals (Glyn-Jones et al., 2015). The pathogenesis of OA involves the destruction of articular cartilage, subchondral osteosclerosis, and the formation of osteophytes (Hunter and Bierma-Zeinstra, 2019). Although morbidity is strongly age-dependent, aging is not the only cause of OA. Many physiological and mechanical factors, including trauma, sex, heredity, obesity, and mechanical stress, play a role in accelerating the progression of this disease, and all of these factors can destroy the synovium and articular cartilage (Morales-Ivorra et al., 2018). Among these pathogenic factors, inflammation is a critical cause of both joint cartilage destruction and the symptoms of OA (Sellam and Berenbaum, 2010). Inflammatory cytokines such as interleukin-1β (IL-1β) can induce the production of catabolic factors, including thrombospondin motifs (ADAMTS) and matrix metalloproteinases (MMPs); inhibit the synthesis of synthetic metabolic factors, such as collagen and proteoglycans; and promote the death of chondrocytes and thus have destructive effects on articular cartilage (Hosseinzadeh et al., 2016). In addition, many metabolic factors, such as hyperlipidemia (Frey et al., 2017; Garcia-Gil et al., 2017) and iron overload (Valentino, 2010; Carroll et al., 2011; Camacho et al., 2016), are also closely associated with OA.

Because chondrocytes are the only cellular constituent of articular cartilage, they maintain the integrity of cartilage by balancing the synthesis and degradation of extracellular matrix (ECM) (Sandell and Aigner, 2001). Therefore, chondrocyte injury is an essential issue in the progression of OA. Previous studies have found that cell necrosis, apoptosis, and autophagic cell death can cause damage to chondrocytes (Jiang et al., 2020). In 2012, Dixon et al. first identified a novel form of cell death whose morphology, biochemistry, and genetics are totally different from those of other forms of regulated cell death and named it ferroptosis (Dixon et al., 2012). According to the Nomenclature Committee on Cell Death, ferroptosis is defined as a form of regulated cell death caused by oxidative disturbance of the intracellular microenvironment that is constitutively controlled by glutathione peroxidase 4 (GPX4), which can be inhibited by iron chelators and lipophilic antioxidants (Galluzzi et al., 2018). Recent studies have shown that GPX4 is a crucial regulatory factor in ferroptosis (Seibt et al., 2019). Acyl-CoA synthetase long-chain family member 4 (ACSL4) is not only a sensitive index used to monitor ferroptosis but also an important contributor to ferroptosis (Yuan et al., 2016; Doll et al., 2017). Lysophosphatidylcholine acyltransferase 3 (lpcat3) causes ferroptosis by regulating arachidonic acid metabolism (Wang and Tontonoz, 2019). LOX15 (15-lipoxygenase) has been suggested to be a central player in ferroptosis and can effectively oxidize diverse phosphatidylethanolamines into ferroptotic signaling molecules facilitated by ACSL4-driven esterification (Doll et al., 2017; Shah et al., 2018). The inhibition of SLC7A11, a subunit of cystine/glutamate antiporter, resulted in the depletion of intracellular glutathione, iron-dependent lipid peroxidation, and subsequent ferroptosis (Lewerenz et al., 2013). As a tumor suppressor, P53 is not only a key regulatory element in apoptosis but also a critical molecule in ferroptosis as it inhibits the production of SLC7A11 (Jiang et al., 2015). Thus far, ferroptosis has been demonstrated to be associated with many degenerative illnesses (such as brain injury, cerebral hemorrhage, Alzheimer’s disease, cancer, stroke, trauma, ischemia-reperfusion injury, Parkinson’s disease, and renal degeneration) (Stockwell et al., 2017). Some studies have confirmed that OA occurs and develops as a result of ferroptosis. For example, lipid peroxidation, abnormal iron metabolism, and mitochondrial dysfunction may be involved in the progression of OA (Yao et al., 2021).

Deferoxamine (DFO) is an effective iron-chelating agent approved by the Food and Drug Administration that is used for the treatment of iron-overload diseases (Yao et al., 2019a). Because iron deposition is an important pathological event in ferroptosis (Dixon et al., 2012; Friedmann Angeli et al., 2019; Hirschhorn and Stockwell, 2019), DFO, as a powerful iron chelator, has been used to inhibit ferroptosis in various disease models (Ma et al., 2016; Bruni et al., 2018). In addition, DFO was reported to reverse ferroptosis in spermatogenic cells by activating nuclear factor E2–related factor 2 (Nrf2), thereby alleviating busulfan-induced male infertility (Zhao et al., 2020). As for treatment of OA, several experiments have shown that DFO suppresses the expression of MMPs induced by IL-1β in chondrocytes stimulated by IL-1β (Jing et al., 2020).

On the basis of these experimental results, we hypothesized that DFO maintains the dynamic balance of cartilage under various harmful conditions and delays the progression of OA by inhibiting the ferroptosis of chondrocytes and activating Nrf2-mediated protection. The role of DFO in reducing the expression of MMPs has been mentioned previously (Jing et al., 2020). Nevertheless, some potential effects of DFO in the development of OA, such as its antioxidant, anti-ferroptotic, and anti-inflammatory effects, are still unclear. In this study, IL-1β was used to simulate the inflammatory response, and erastin was used to induce chondrocyte ferroptosis *in vitro*. In addition, we induced OA *in vivo* by performing destabilized medial meniscus (DMM) surgery of the mouse knee joints. We aimed to study the regulatory effects of DFO on ferroptosis and the Nrf2 signaling pathway in chondrocytes and ascertain whether DFO can protect OA chondrocytes and cartilage.

## Methods and Materials

### Regents

DFO (USA, D9533) was purchased from Sigma-Aldrich. Recombinant mouse IL-1β (# 401-ML) and dimethylsulfoxide (DMSO) were purchased from R&D Systems (Minneapolis, USA). Erastin (USA, S7242) was obtained from Selleck and diluted in DMSO. An ROS assay kit (China, S0033) was provided by Beyotime. A C11 BODIPY Lipid Peroxidation Sensor (USA, D3861) was obtained from Thermo Fisher. A Micro Malondialdehyde (MDA) Assay Kit (China, BC0025) was purchased from Solarbio. A Micro Iron Concentration Assay Kit (China, TC1016) was provided by Leagene Biotechnology. Antibodies against GPX4 (UK, ab125066, diluted 1:5,000), SLC7A11 (UK, ab175186, diluted 1:5,000), ACSL4 (UK, ab155282, diluted 1:10,000), and P53 (UK, ab131442, diluted 1:1,000) were purchased from Abcam. Antibodies against Nrf2 (USA, 16396-1-AP, diluted 1:1,000), HO-1 (USA, 10701-1-AP, diluted 1:1,000), collagen II (USA, 28459-1-AP, diluted 1:2,000), MMP13 (USA, 18165-1-AP, diluted 1:2,000) and GAPDH (USA, 60004-1-Ig, diluted 1:1,000) were purchased from Proteintech. Antibodies against LOX15 (China, A6865, diluted 1:1,000) and lpcat3 (China, A17604, diluted 1:1,000) were purchased from Abclonal.

### Isolation and Culture of Murine Chondrocytes

Chondrocytes were isolated from 5-day-old C57BL/6J mice and cultured as described before (Sun et al., 2019). All animal experimental procedures were approved by the Experimental Animal Ethics Committee of Tongji Medical College, Huazhong University of Science and Technology, Wuhan, China. In brief, cartilage was removed from the knee joints and dissected into pieces. The cartilage was digested with 0.25% trypsin for 30 min and 0.25% type 2 collagenase for 6 h. The primary chondrocytes were resuspended and cultured in DMEM/F12 medium containing 10% fetal bovine serum, 1% penicillin, and 1% streptomycin sulfate at 37°C in a humidified atmosphere of 5% CO_2_. Chondrocytes at the first and second passages were used in the experiments.

### Western Blot Analysis

Chondrocytes were seeded in six-well plates at a density of 5 × 10^5^ cells per well and adhered for 48 h. Then, Western blot analysis was performed as described before (Yao et al., 2019b). Briefly, cells from different treatment groups were harvested with RIPA lysis buffer (Boster, China, AR0102) containing 1% phenylmethylsulfonyl fluoride and 1% phosphatase inhibitor cocktail for 30 min on ice. The extract was collected and centrifuged at 12,000 × g and 4°C for 30 min. Then, the supernatant was collected, and the protein concentration of each sample was detected with a BCA assay kit (Boster, China, AR0146). Samples containing a quantity of proteins were separated by sodium dodecyl sulfate–polyacrylamide gel electrophoresis (SDS-PAGE) and transferred to PVDF membranes (Millipore, USA). After blocking with 5% skim milk at room temperature for 1 h, the membranes were incubated overnight with specific primary antibodies at 4°C and then incubated with secondary antibodies for 1 h at room temperature. Protein bands were visualized using chemiluminescence reagent (Boster, China), and images were obtained with a Bio-Rad scanner (Hercules, CA, USA).

### Toluidine Blue Staining

Toluidine blue staining was used to observe the morphology of chondrocytes according to the manufacturer’s instructions. Briefly, chondrocytes were seeded in 35-mm plastic dishes (10^5^ cells per well). At 80% confluency, cells were treated with IL-1β (10 ng/ml), erastin (5 µM), or/and DFO (50, 100, and 200 µM) for 24 h. Then, the chondrocytes were fixed with 4% paraformaldehyde for 15 min at room temperature after washing with PBS. Subsequently, the cells were gently stained with toluidine blue for 24 h. Finally, excess dye was removed by washing with PBS three times for 5 min each. Then, the morphological characteristics of chondrocytes were observed using a microscope (Evos Fl Auto, Life Technologies, USA).

### Cell Viability Assay

Chondrocyte viability was detected using the Cell Counting Kit-8 (CCK-8) (Boster, China, AR1160) assay. Chondrocytes were plated into 96-well plates at a density of 3,000 cells per well with five replicate wells. After adherence for 24 h, the cells were treated with DFO at a series of concentrations alone or in combination with IL-1β or erastin for 24 h. After the medium had been removed, 100 μl of a 10% CCK-8 solution was added to each well and incubated at 37°C away from light for 1 h. The absorbance at 450 nm was measured using a microplate reader (Thermo Fisher Scientific, Vantaa, Finland).

### Detection of Intracellular ROS and Lipid ROS

Intracellular ROS and lipid ROS levels were measured with the fluorescent probes DCFH-DA and C11-BODIPY according to the manufacturer’s instructions. In brief, chondrocytes were first seeded in six-well plates at a density of 3 × 10^5^ cells per well. After 24 h, the chondrocytes from different treatment groups were washed with PBS three times and treated with 10 μM DCFH-DA or 10 μM C11 BODIPY for 30 min at 37°C in the dark. After incubation, the cells were washed with PBS and observed under a fluorescence microscope (EVOS FL Auto, Life Technologies, USA). C11-bodipy was excited at 561 nm to show the red fluorescence intensity associated with the unoxidized dye and excited at 488 nm to show the green fluorescence intensity associated with the oxidized dye.

### Malondialdehyde Assay

The relative MDA concentration in cell lysates was assessed using an MDA assay kit (Solarbio, China) according to the manufacturer’s instructions. Briefly, the reaction of the MDA in the sample with thiobarbituric acid (TBA) generated an MDA-TBA adduct. The MDA-TBA adduct was quantified fluorometrically (at an excitation wavelength of 532 nm and emission wavelength of 553 nm).

### Fe^2+^ Assay

The determination of intracellular ferrous iron level (Fe^2+^) uses the iron assay kit (#ab83366, Abcam). First, samples were collected, washed with cold PBS, and homogenized in iron assay buffer; then a 5-µl iron reducer was added in to the Standard wells; and 5 µl of Assay Buffer was added to each sample, mixed, and incubated 30 min. Last, a 100-µl iron probe was added, mixed, and incubated for 1 h, and the content was immediately measured on a colorimetric microplate reader (the absorbance is 593 nm).

### Knockdown of Nrf2 by Small Interfering RNA

Specific small interfering RNA (siRNA) targeting the mouse Nrf2 gene was chemically synthesized by RiboBio (Guangzhou, China) and transfected into cells using Lipofectamine 3,000 transfection reagent following the manufacturer’s instructions (Thermo Fisher, UT, USA). The Nrf2 siRNA sense strand sequence was reported in our previous article (Sun et al., 2019): 5′-CGACAGAC CCTCCATCTA-3′.

### Animal Experiment

Eight-week-old male C57BL/6 mice were supplied by the Experimental Animal Centre of Tongji Hospital, and the Institutional Animal Care and Use Committee of Tongji Hospital (Wuhan, China) approved all animal experiments. To establish an experimental OA model, DMM was surgically induced on the right knee of the mice, which were anesthetized by intraperitoneal injection of pentobarbital (35 mg/kg). Fifty-six mice were randomly divided into seven groups (*n* = 8 for each group): the sham group, DMM group, DMM + DFO (10 mg/kg) group, DMM + DFO (100 mg/kg) group, erastin group, erastin (5 mg/kg) + DFO (10 mg/kg) group, and erastin (5 mg/kg) + DFO (100 mg/kg) group. The mice were intra-articularly injected with DFO (10 or 100 mg/kg). Mice in the sham surgery group and DMM group were intra-articularly injected with the same volume of vehicle. The injection was repeated twice a week for 8 consecutive weeks. Eight weeks later, the mice were sacrificed, and the knee joints were collected and fixed in 4% paraformaldehyde for further experiments.

### Histological and Immunohistochemical Staining

After the mice had been euthanized, all right knee joints were excised, fixed in 4% paraformaldehyde for 24 h, decalcified with a 10% EDTA solution for 2 weeks and embedded in paraffin wax. The specimens were sectioned to a thickness of 5 μm in the sagittal plane and then stained with safranin O/fast green. The progression of OA was evaluated using the Osteoarthritis Research Society International (OARSI) score in a blinded manner (Bannuru et al., 2019). After they were deparaffinized and rehydrated, the sections were blocked in BSA containing 0.1% Triton X-100 for 1 h for histochemistry. The sections were then incubated with anti-ACSL4, anti-MMP13, or anti–collagen II antibodies, followed by goat anti-rabbit secondary antibody; colored with DAB; and counterstained with hematoxylin. Images were captured by fluorescence microscopy (EVOS FL Auto, Life Technologies, USA).

### Statistical Analyses

Data are expressed as the mean ± SD for independent experiments and were analyzed with GraphPad Prism 7 (GraphPad Software, San Diego, CA, USA). One-way analysis of variance followed by *post hoc* comparison with the Bonferroni’s test and Student’s t-test was used to compare all groups studied. *p* < 0.05 was used to indicate statistical significance.

## Results

### Effect of DFO on Chondrocyte Viability

**FIGURE 1:**
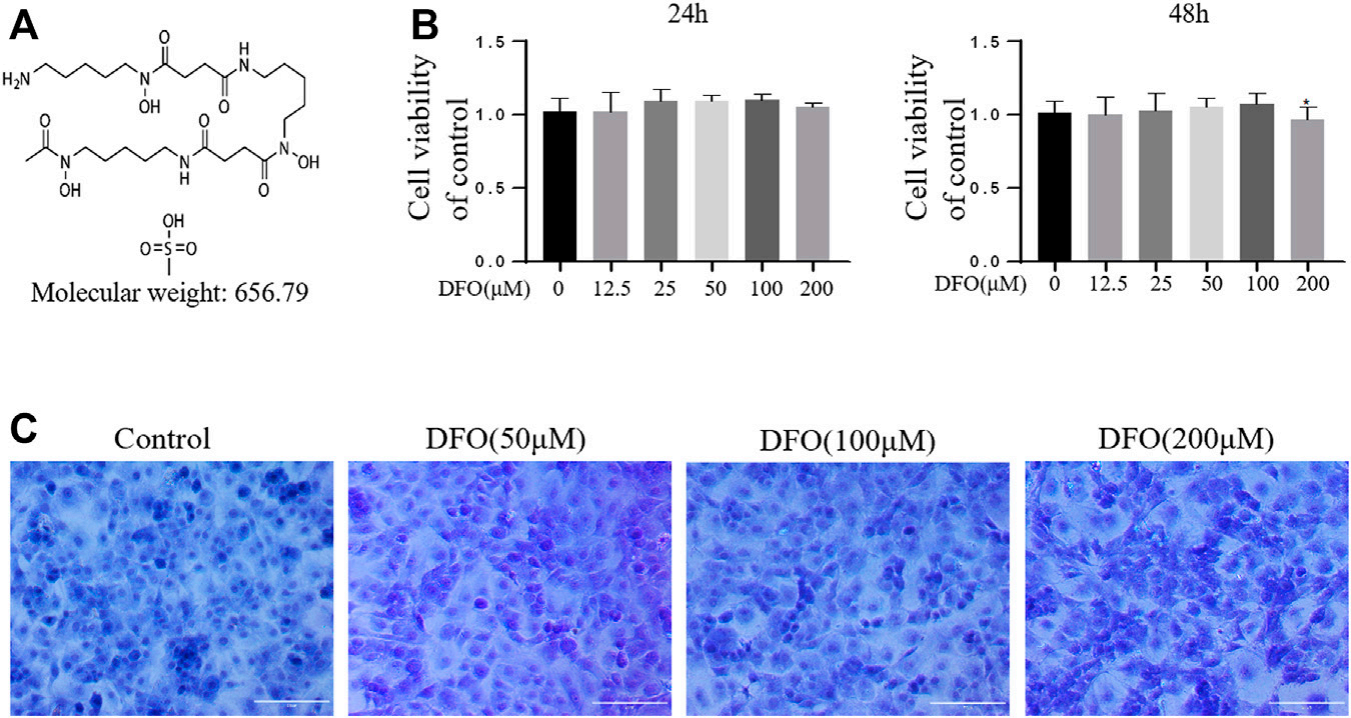
Effect of DFO on the chondrocyte viability. **(A)** Chemical structure of molecular weight of DFO. **(B)** The cytotoxic effect of DFO (12.5, 25, 50, 100, and 200 μM) on the chondrocytes was determined for 24 and 48 h. The columns represent the means ± SD. *p < 0.05 versus the control, *n* = 5. **(C)** Chondrocyte morphology is revealed by toluidine blue staining.

The chemical structure of DFO is shown in Figure 1A. The cytotoxicity of 12.5, 25, 50, 100, and 200 μM DFO was assessed in mouse chondrocytes for 24 and 48 h (Figure 1B). DFO (200 μM) treatment for 48 h repressed cell viability (*p* < 0.05), indicating that DFO at concentrations below 100 μM had no significant cytotoxicity. Therefore, in our experiments, chondrocytes were treated with 50 and 100 μM DFO. Chondrocyte morphology was observed using toluidine blue staining. Compared with the control group, the chondrocytes treated with DFO (50, 100, and 200 μM) showed little loss of coloration (Figure 1C).

### IL-1β Induced Ferroptosis in Chondrocytes, Which Could Be Rescued by Ferrostatin-1

**FIGURE 2:**
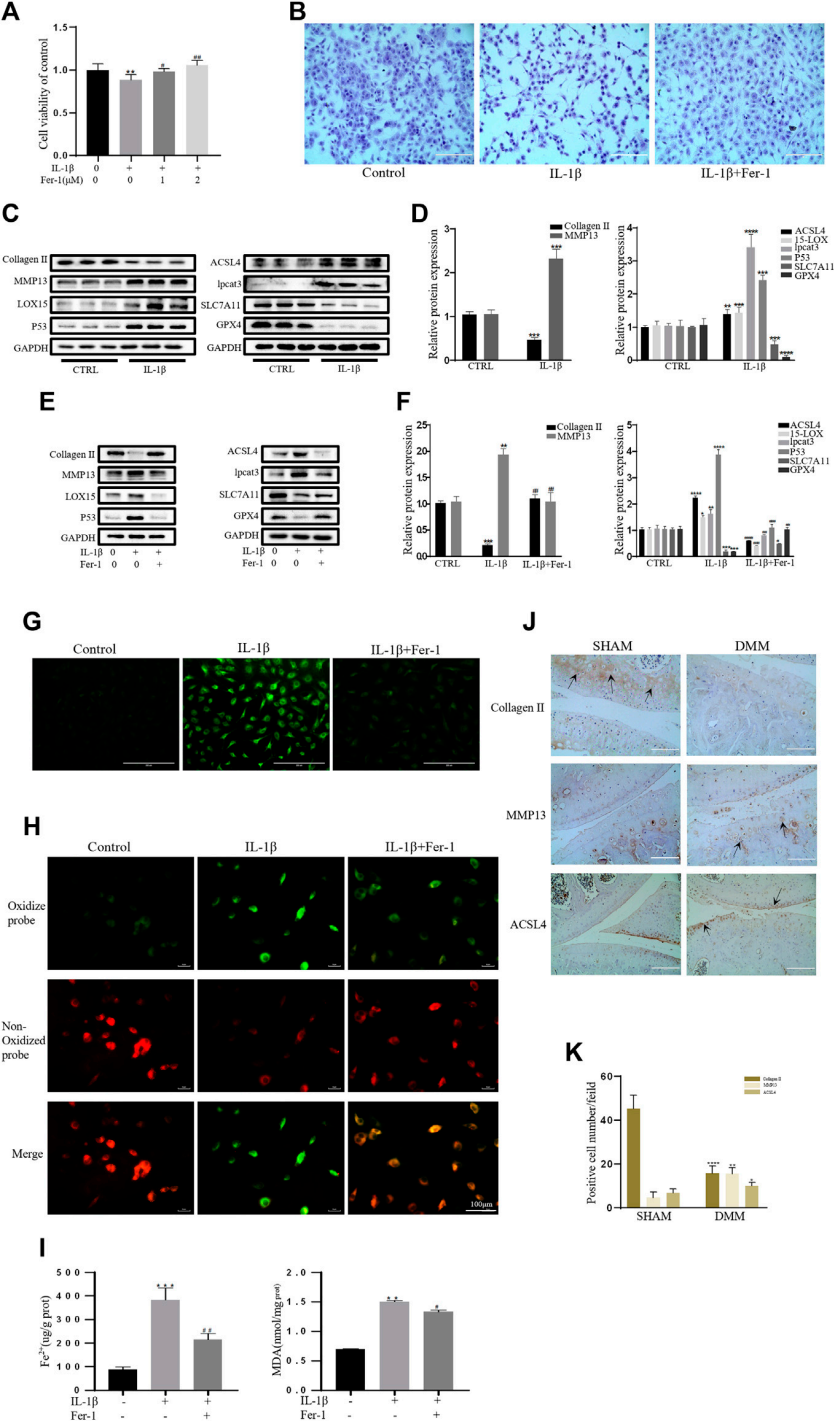
IL-1β induced MDA, ROS, and lipid–ROS accumulation and ferroptosis-related protein expression changes in chondrocytes, which can be rescued by Ferrostatin-1. **(A,B)** Cell viability determined by CCK-8 assay and toluidine blue staining. **(C,D)** The protein expression levels of collagen Ⅱ, MMP13, ACSL4, LOX15, lpcat3, P53, and SLC7A11 GPX4 when treated by IL-1β (10 ng/ml) were detected by Western blot, and band density ratios of collagen Ⅱ, MMP13, ACSL4, LOX15, lpcat3, P53, and SLC7A11 GPX4 to GAPDH in the Western blots were quantified by densitometry (*n* = 3). **(E,F)** The protein expression levels of collagen Ⅱ, MMP13, ACSL4, LOX15, lpcat3, P53, and SLC7A11 GPX4 when treated by IL-1β (10 ng/ml) with fer-1 (1 μM) or equal volume of DMSO were detected by Western blot, and band density ratios of collagen Ⅱ, MMP13, ACSL4, LOX15, lpcat3, P53, and SLC7A11 GPX4 to GAPDH in the Western blots were quantified by densitometry (*n* = 3). **(G)** Intracellular ROS level detected by DCFH-DA fluorescent probe (scale bar: 200 µm). **(H)** Intracellular lipid-ROS level detected by C11 BODIPY fluorescent probe (scale bar: 100 µm). Red, reduced form of C11-BODIPY; green, oxidized form of C11-BODIPY. **(I)** The intracellular level of MDA and Fe^2+^ was determined using the MDA assay kit and iron assay kit (*n* = 3). **(J)** The collagen Ⅱ, MMP13, and ACSL4 expression levels in the cartilage samples were measured using immunohistochemistry staining. Dotted arrows indicate positive cells for MMP13 and ACSL4 and positive staining of collagen Ⅱ (scale bar: 100 µm). **(K)** Quantification of MMP13- and ACSL4-positive cells and collagen Ⅱ–positive staining *in vivo*. *p < 0.05 versus control or the sham group, **p < 0.01 versus control or the sham group, ***p < 0.001 versus control or the sham group, ****p < 0.0001 versus control or the sham group, #*p* < 0.05 versus IL-1β treated group, ##*p* < 0.01 versus IL-1β–treated group, and ###*p* < 0.001 versus IL-1β–treated group. Error bars represent SD.

As a specific effective inhibitor of ferroptosis, ferrostatin-1 (Fer-1) can eliminate alkoxyl radicals and other products of rearrangement induced by ferrous iron from lipid hydroperoxides (Miotto et al., 2020). The ferroptosis of chondrocytes treated with IL-1β with or without Fer-1 co-treatment was tested. The CCK-8 assay and toluidine blue staining showed that IL-1β was obviously cytotoxic to the chondrocytes, but this cytotoxicity was partially mitigated by Fer-1 (Figures 2A,B). In our experiments, the protein expression levels of SLC7A11, GPX4, P53, ACSl4, LOX15, and lpcat3 and the concentrations of MDA and Fe^2+^ in chondrocytes were utilized as markers of ferroptosis, and the protein expression levels of collagen II and MMP13 were utilized as markers of OA. Western blot analysis showed that IL-1β decreased the protein level of collagen II and increased the production of MMP13 in chondrocytes. The results also showed that the expression of SLC7A11 and GPX4 was inhibited, and the protein levels of ACSl4, LOX15, P53, and lpcat3 were upregulated in chondrocytes stimulated with IL-1β (Figures 2C,D). However, Fer-1 alleviated the increased expression of MMP13, ACSL4, LOX15, P53, and lpcat3 and restored the expression of collagen II, SLC7A11, and GPX4 (Figure 2E). Densitometry was used to detect the collagen II and MMP13, GPx4, SLC7A11, p53, ACSl4, LOX15, and lpcat3 band density relative to that of GAPDH. The difference in relative protein band density was statistically significant (Figure 2F). In addition, the results of DCFH-DA and C11 BODIPY fluorescence analyses showed that IL-1β treatment induced the accumulation of both ROS and lipid ROS in chondrocytes, which could be alleviated by Fer-1 treatment (Figures 2G,H). Compared with the control group, IL-1β–stimulated chondrocytes produced more MDA and Fe^2+^, and Fer-1 curbed MDA and Fe^2+^ accumulation in the chondrocytes (Figure 2I). Immunohistochemical analysis of mouse knee joints showed that DMM induced cartilage collagen II loss and significantly increased MMP13- and ACSl4-positive cells (Figures 2J,K). These results suggested that IL-1β can simultaneously induce the expression of ferroptosis-related proteins and OA-like changes in chondrocytes.

### Erastin Initiated Inflammatory Responses and ECM Degradation in Chondrocytes, Which Was Alleviated by Fer-1

**FIGURE 3:**
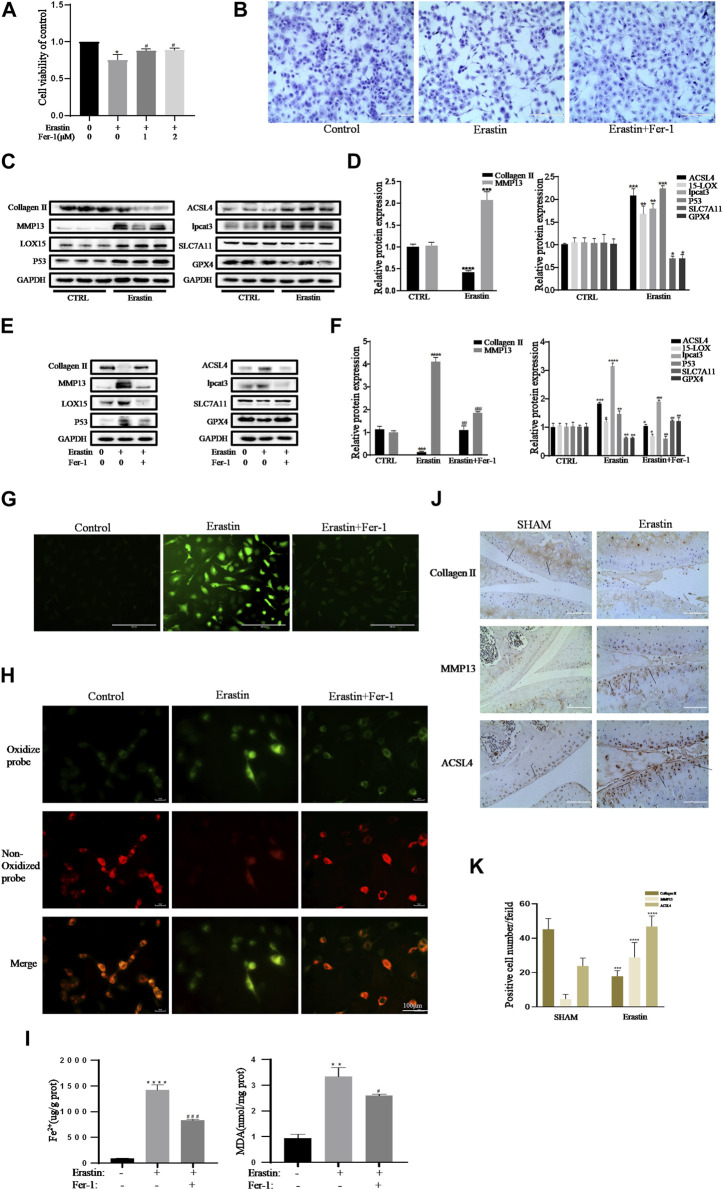
Erastin initiated inflammation responses and ECM degradation in chondrocytes that could be alleviated by Ferrostatin-1. **(A,B)** Cell viability determined by CCK-8 assay and toluidine blue staining. **(C,D)** The protein expression levels of collagen Ⅱ, MMP13, ACSL4, LOX15, lpcat3, P53, and SLC7A11 GPX4 when treated by erastin (5 μM) were detected by Western blot, and band density ratios of collagen Ⅱ, MMP13, ACSL4, LOX15, lpcat3, P53, and SLC7A11 GPX4 to GAPDH in the Western blots were quantified by densitometry (*n* = 3). **(E,F)** The protein expression levels of collagen Ⅱ, MMP13, ACSL4, LOX15, lpcat3, P53, and SLC7A11 GPX4 when treated by Erastin (5 μM) with fer-1 (1 μM) or equal volume of DMSO were detected by Western blot, and band density ratios of collagen Ⅱ, MMP13, ACSL4, LOX15, lpcat3, P53, and SLC7A11 GPX4 to GAPDH in the Western blots were quantified by densitometry (*n* = 3). **(G)** Intracellular ROS level detected by DCFH-DA fluorescent probe (scale bar: 200 µm). **(H)** Intracellular lipid-ROS level detected by C11 BODIPY fluorescent probe (scale bar: 100 µm). Red, reduced form of C11-BODIPY; green, oxidized form of C11-BODIPY. **(I)** The intracellular level of MDA and Fe^2+^ was determined using the MDA assay kit and iron assay kit (*n* = 3). **(J)** The collagen Ⅱ, MMP13, and ACSL4 expression levels in the cartilage samples were measured using immunohistochemistry staining. Dotted arrows indicate positive cells for MMP13 and ACSL4 and positive staining of collagen Ⅱ (scale bar: 100 µm). (K) Quantification of MMP13- and ACSL4-positive cells and collagen Ⅱ–positive staining in vivo. *p < 0.05 versus control or the sham group, **p < 0.01 versus control or the sham group, ***p < 0.001 versus control or the sham group, ****p < 0.0001 versus control or the sham group, #*p* < 0.05 versus IL-1β–treated group, ##*p* < 0.01 versus IL-1β–treated group, and ###*p* < 0.001 versus IL-1β–treated group. Error bars represent SD.

Collagen II secreted by chondrocytes is the principal component of the ECM. MMP13, the main ECM-degrading enzyme, is significantly overexpressed in articular cartilage in OA (Vincenti and Brinckerhoff, 2001; Miotto et al., 2020). Therefore, a decrease in collagen II expression and an increase in MMP13 expression are important contributors and biomarkers of chondrocyte lesions in OA. Erastin is an effective classic inducer of ferroptosis (Dixon et al., 2012). The CCK-8 assay and toluidine blue staining showed that erastin had obvious cytotoxic effects on chondrocytes, but Fer-1 partially alleviated this cytotoxicity (Figures 3A,B). Treatment with erastin decreased the protein levels of collagen II, SLC7A11, and GPX4 and promoted the expression of MMP13, ACSl4, LOX15, lpcat3, and P53 in chondrocytes (Figures 3C,D). Fer-1 suppressed the decreases in collagen II, SLC7A11, and GPX4 and attenuated the increases in MMP13, ACSL4, LOX15, P53, and lpcat3 (Figure 3E). There was a statistically significant difference in the band density measured by densitometry of every protein relative to that of GAPDH (Figure 3F). The fluorescence results showed that intracellular ROS and lipid ROS accumulated after treatment with erastin (as reflected by the green fluorescence intensity). Fer-1 decreased the levels of ROS and lipid ROS in chondrocytes (Figures 3G,H). Compared with the control group, erastin stimulated chondrocytes to produce more MDA and Fe^2+^, and Fer-1 reduced MDA and Fe^2+^ accumulation in chondrocytes (Figure 3I). Immunohistochemical analysis of mouse knee joint samples showed that intra-articular injection of erastin induced cartilage collagen II loss and significantly increased MMP13- and ACSl4-positive cells (Figures 3J,K). These results indicated that erastin can also induce OA-like changes in chondrocytes and promote OA development while it induces chondrocyte ferroptosis.

### DFO Alleviated the Inflammatory Response and ECM Degradation in Chondrocytes Induced by IL-1β by Inhibiting Chondrocyte Ferroptosis

**FIGURE 4:**
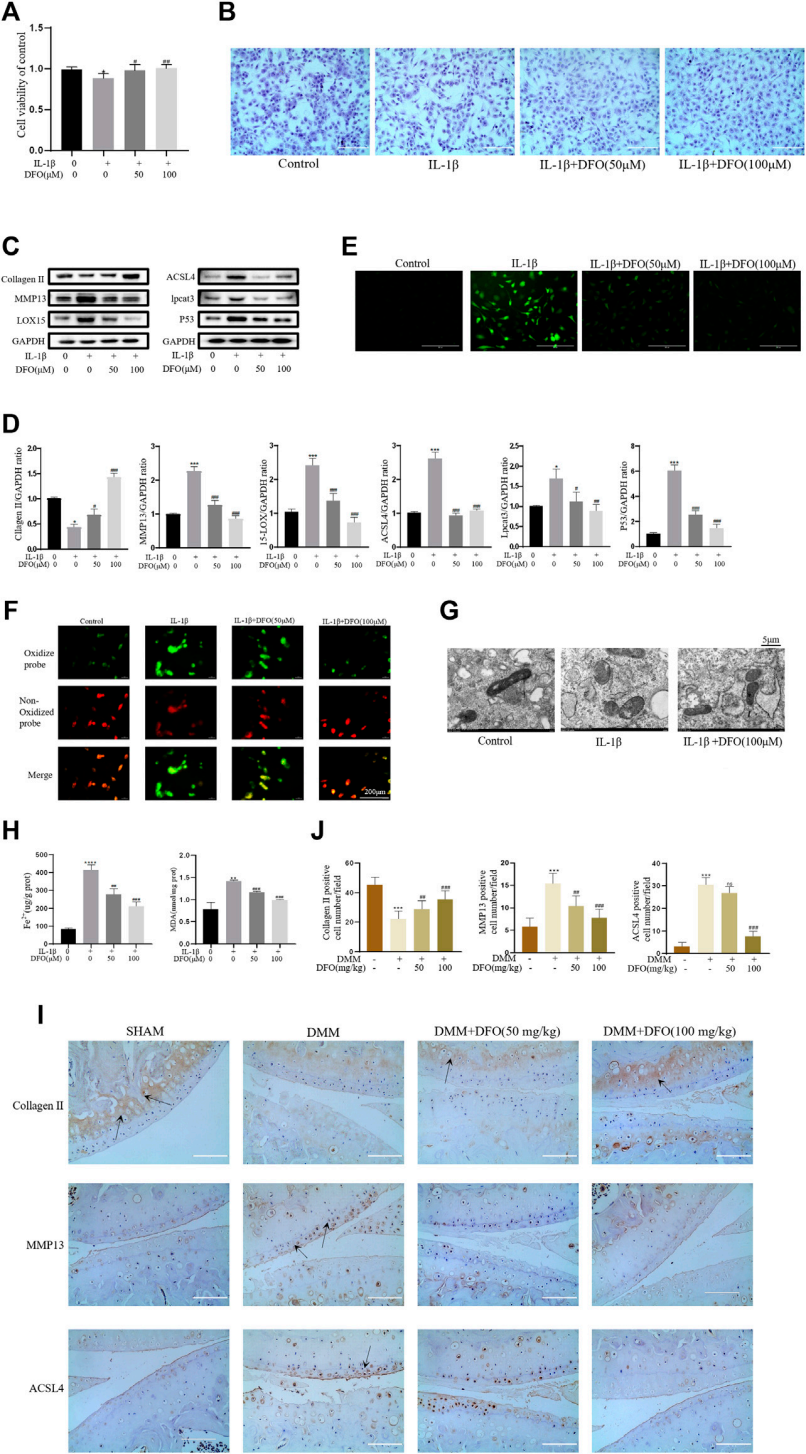
DFO attenuated the inflammatory response and ECM degradation of chondrocytes induced by IL-1β *via* inhibiting chondrocytes ferroptosis. **(A,B)** Cell viability determined by CCK-8 assay and toluidine blue staining. **(C,D)** The protein expression levels of collagen Ⅱ, MMP13, ACSL4, LOX15, lpcat3, and P53 when treated by IL-1β (10 ng/ml) with 50 and 100 μM DFO or equal volume of DMSO were detected by Western blot, and band density ratios of collagen Ⅱ, MMP13, ACSL4, LOX15, lpcat3, and P53 to GAPDH were quantified by densitometry (*n* = 3). **(E)** Intracellular ROS level detected by DCFH-DA fluorescent probe (scale bar: 200 µm). **(F)** Intracellular lipid-ROS level detected by C11 BODIPY fluorescent probe (scale bar: 200 µm). Red, reduced form of C11-BODIPY; green, oxidized form of C11-BODIPY. **(G)** The ultrastucture of mitochondria observed via transmission electron microscopy (scale bar: 5 µm). **(H)** The intracellular level of MDA and Fe^2+^ was determined using the MDA assay kit and iron assay kit (*n* = 3). **(I)** The collagen Ⅱ, MMP13, and ACSL4 expression levels in the cartilage samples were measured using immunohistochemistry staining. Dotted arrows indicate positive cells for MMP13 and ACSL4 and positive staining of collagen Ⅱ (scale bar: 100 µm). **(J)** Quantification of MMP13- and ACSL4-positive cells and collagen Ⅱ–positive staining *in vivo*. *p < 0.05 versus control or the sham group, **p < 0.01 versus control or the sham group, ***p < 0.001 versus control or the sham group, #*p* < 0.05 versus IL-1β–treated group or the DMM group, ##*p* < 0.01 versus IL-1β–treated group or the DMM group, ###*p* < 0.001 versus IL-1β–treated group or the DMM group or the DMM group, ####*p* < 0.0001 versus IL-1β–treated group or the DMM group or the DMM group. Error bars represent SD.

We tested whether DFO could exert beneficial effects on chondrocytes induced by IL-1β. The CCK-8 assay and toluidine blue staining showed that DFO partially attenuated the cytotoxic effects of IL-1β on chondrocytes (Figures 4A,B). Western blot analysis showed that DFO attenuated the upregulation of MMP13 expression and rescued the downregulation of collagen II expression induced by IL-1β. In addition, DFO blocked the expression of IL-1β–induced proteins related to ferroptosis (Figures 4C,D). Our data showed that DFO suppressed the accumulation of ROS and lipid ROS in chondrocytes treated with IL-1β and further suppressed the increased production of MDA and Fe^2+^ stimulated by IL-1β (Figures 4E,F,H). To verify the damaging effect of IL-1β on mitochondria *in vitro* and to determine whether DFO could improve it, we observed the ultrastructure of mitochondria by transmission electron microscopy (TEM). As shown in Figure 4G, DFO significantly improved IL-1β–induced mitochondrial damage in chondrocytes. After DFO treatment, the mitochondrial membrane was intact, and the mitochondria were narrower with an increased number of cristae compared with mitochondria in the IL-1β group (Figure 4G). Immunohistochemical analysis showed that compared with that in the DMM group, intra-articular injection of DFO significantly decreased the expression of MMP13 and ACSL4 and restored collagen II loss in articular cartilage (Figures 4I,J). These results indirectly demonstrate that DFO can modulate the IL-1β–induced inflammatory response and maintain cartilage homeostasis by inhibiting chondrocyte ferroptosis.

### DFO Alleviated Chondrocyte Ferroptosis and OA Progression Induced by Erastin

**FIGURE 5:**
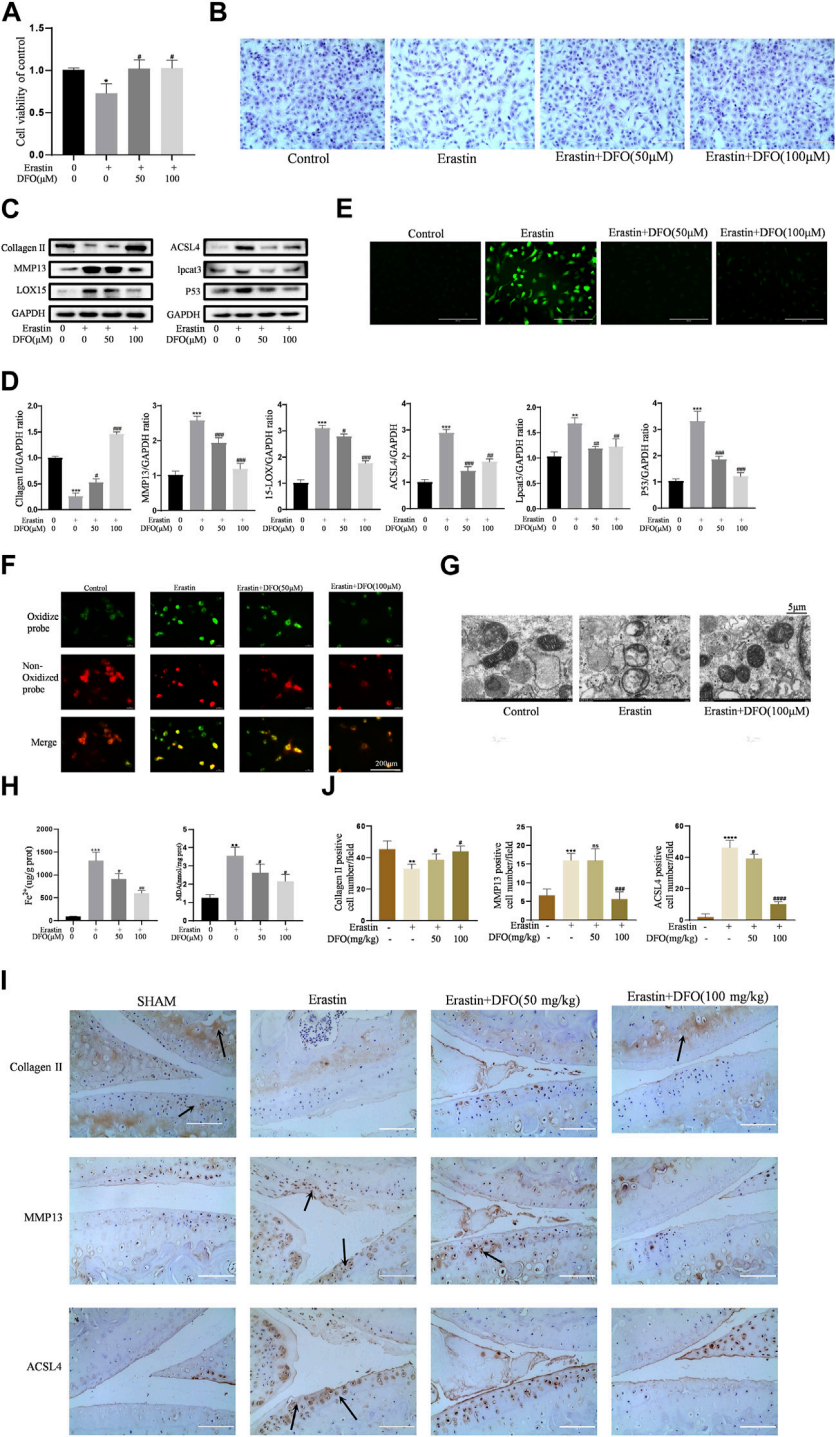
DFO alleviated chondrocytes ferroptosis and OA progress induced by erastin. **(A,B)** Cell viability determined by CCK-8 assay and toluidine blue staining. **(C,D)** The protein expression levels of collagen Ⅱ, MMP13, ACSL4, LOX15, lpcat3, and P53 when treated by Erastin (5 μM) with 50 and 100 μM DFO or equa volume of DMSO were detected by Western blot, and band density ratios of collagen Ⅱ, MMP13, ACSL4, LOX15, lpcat3, and P53 to GAPDH in the Western blots were quantified by densitometry (*n* = 3). **(E)** Intracellular ROS level detected by DCFH-DA fluorescent probe (scale bar: 200 µm). **(F)** Intracellular lipid-ROS level detected by C11 BODIPY fluorescent probe (scale bar: 200 µm). Red, reduced form of C11-BODIPY; green, oxidized form of C11-BODIPY. **(G)** The ultrastucture of mitochondria observed via transmission electron microscopy (scale bar: 5 µm). **(H)** The intracellular level of MDA and Fe2+ was determined using the MDA assay kit and iron assay kit *n* = 3). **(I)** The collagen Ⅱ, MMP13, and ACSL4 expression levels in the cartilage samples were measured using immunohistochemistry staining. Dotted arrows indicate positive cells for MMP13 and ACSL4 and positive staining of collagen Ⅱ (scale bar: 100 µm). **(J)** Quantification of MMP13- and ACSL4-positive cells and collagen Ⅱ–positive staining in vivo. *p < 0.05 versus control or the sham group, **p < 0.01 versus control or the sham group, ***p < 0.001 versus control or the sham group, ****p < 0.0001 versus control or the sham group, #*p* < 0.05 versus IL-1β–treated group or the DMM group, ##*p* < 0.01 versus IL-1β–treated group or the DMM group, ###*p* < 0.001 versus IL-1β–treated group or the DMM group, and ####*p* < 0.0001 versus IL-1β–treated group or the DMM group. Error bars represent SD.

To further verify the effect of DFO on chondrocyte ferroptosis, we used DFO to treat erastin-treated chondrocytes. We determined whether DFO can exert beneficial effects on erastin-treated chondrocytes. The CCK-8 assay and toluidine blue staining showed that DFO partially attenuated the cytotoxic effects of erastin on chondrocytes (Figures 5A,B). Western blot analysis showed that DFO attenuated the upregulation of MMP13 protein expression and downregulation of collagen II expression induced by erastin. In addition, DFO blocked the erastin-induced increase in ferroptosis-related protein expression (Figures 5C,D). Our results showed that DFO suppressed the accumulation of ROS and lipid ROS in chondrocytes treated with erastin (Figures 5E,F). The TEM results showed that DFO significantly improved the structural changes in mitochondria caused by erastin, including their irregular rearrangement, swelling, vacuum bubbles, and disappearance of the mitochondrial ridge (Figure 5G). Compared with the control treatment, erastin stimulated chondrocytes to produce more MDA and Fe^2+^, and DFO reduced MDA and Fe^2+^ accumulation in chondrocytes (Figure 5H). The results of immunohistochemical analysis of experimental mouse joint samples showed that intra-articular injection of DFO significantly decreased the expression of MMP13 and ACSL4 and rescued collagen II loss in articular cartilage induced by erastin (Figures 5I,J). These results indirectly demonstrate that DFO can modulate erastin-induced chondrocyte ferroptosis and the inflammatory response to further maintain cartilage homeostasis.

### Nrf2 Signaling Mediated the Protective Effects of DFO on Chondrocytes Induced by IL-1β

**FIGURE 6:**
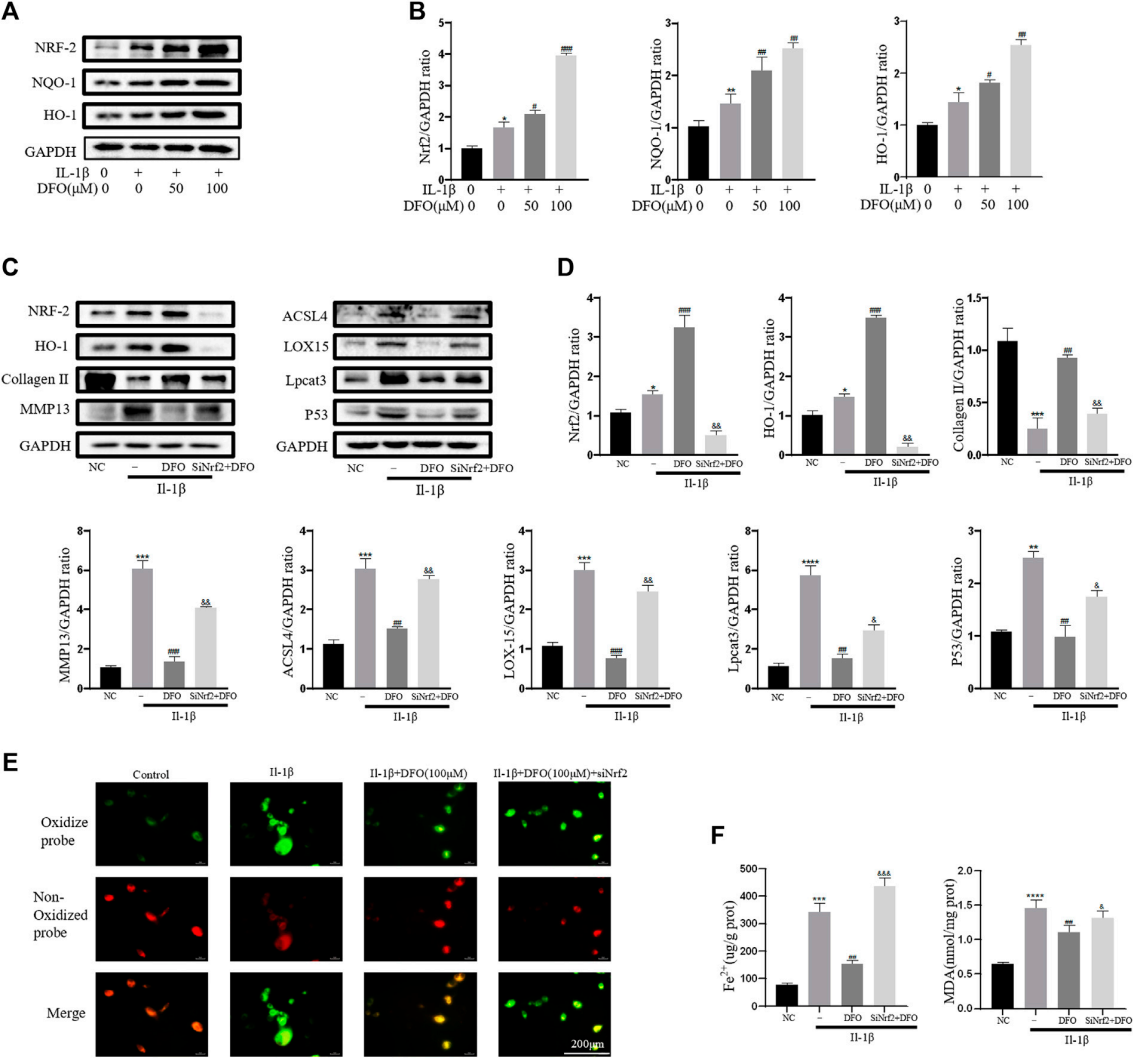
Nrf2 signaling mediated protective effects of DFO on OA chondrocyte. **(A,B)** The protein expression levels of Nrf2, HO-1, and NQO-1 when treated by IL-1β (10 ng/ml) with 50 and 100 μM DFO or equal volume of DMSO were detected by Western blot, and band density ratios of Nrf2, HO-1, and NQO-1 to GAPDH in the Western blots were quantified by densitometry (*n* = 3). **(C,D)** The protein levels of Nrf2, HO-1, collagen Ⅱ, MMP13, ACSL4, LOX15, lpcat3, and P53 when treated by IL-1β (10 ng/ml) with si-Nrf2 or negative control were detected by Western blot, and band density ratios of Nrf2, HO-1, collagen Ⅱ, MMP13, ACSL4, LOX15, lpcat3, and P53 to GAPDH in the Western blots were quantified by densitometry (*n* = 3). **(E)** Intracellular lipid-ROS level detected by C11 BODIPY fluorescent probe (scale bar: 200 µm). Red, reduced form of C11-BODIPY; green, oxidized form of C11-BODIPY. **(F)** The intracellular level of MDA and Fe^2+^ was determined using the MDA assay kit and iron assay kit (*n* = 3). *p < 0.05 versus control, **p < 0.01 versus control, ***p < 0.001 versus control, ****p < 0.0001 versus control, #*p* < 0.05 versus IL-1β–treated group, ##*p* < 0.01 versus IL-1β–treated group, ###*p* < 0.001 versus IL-1β–treated group, &*p* < 0.05 versus IL-1β+DFO group, &&*p* < 0.01 versus IL-1β+DFO group, &&&*p* < 0.001 versus IL-1β+DFO group, and &&&&*p* < 0.0001 versus IL-1β+DFO group. Error bars represent SD.

As an important cellular antioxidant molecule [40], Nrf2 and its downstream effectors, such as HO-1 and NQO-1, are considered key factors in mitigating lipid peroxidation and ferroptosis [41]. When activated, Nrf2 moves from the cytoplasm to the nucleus and then activates target antioxidant enzyme genes by binding the antioxidant response element [42]. In the current study, IL-1β treatment slightly elevated the expression levels of Nrf2, HO-1, and NQO-1 (Figure 6A). The differences in band densities for the proteins were statistically significant (Figure 6B). These results suggested that DFO activated the Nrf2 antioxidant system in chondrocytes by promoting the expression and translocation of Nrf2. Furthermore, knockdown of Nrf2 with siRNA was carried out as described in our previous study [34]. Western blot analysis showed that silencing Nrf2 decreased the beneficial effect of DFO on chondrocytes treated with IL-1β (Figures 6C,D). In addition, C11-BODIPY staining suggested that Nrf2 silencing increased the amount of Lipid-ROS suppressed by DFO (Figure 6E). Nrf2 silencing also weakened the cumulative inhibitory effect of DFO on MDA and Fe^2+^ in chondrocytes (Figure 6F). Together, these data suggest that the Nrf2 antioxidant system plays a critical role in DFO-mediated alleviation of the inflammatory response, ECM destruction, and chondrocyte ferroptosis induced by IL-1β.

### DFO Attenuated Cartilage Degradation *in Vivo*

**FIGURE 7:**
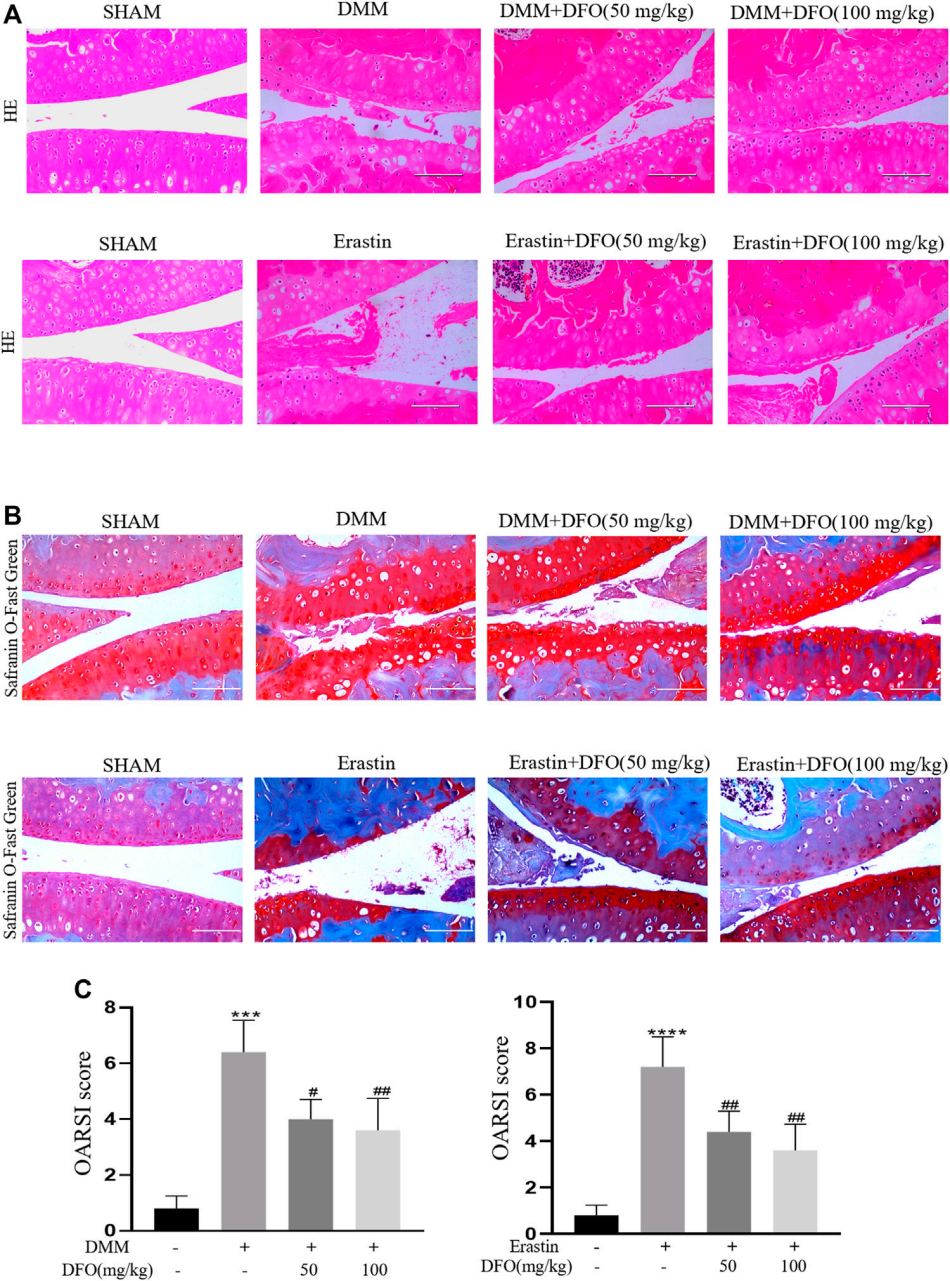
Effect of DFO on the cartilage degradation *in vivo*. Mice received intra-articular injection of DFO (50 or 100 mg/kg) or erastin (5 mg/kg) or vehicle (saline) for 8 weeks after DMM surgery. **(A,B)** Cartilage degradation was histologically evaluated by hematoxylin-eosin staining and safranin O staining/fast green (Scale bar: 100 μm). **(C,D)** The progression of OA was evaluated using the OARSI scores. ***p < 0.0001 versus the sham group, ****p < 0.0001 versus the sham group, #*p* < 0.05 versus the DMM group, and ##*p* < 0.01 versus the DMM group, *n* = 8.

To explore the protective effects of DFO on OA development, model mice were subjected to DMM surgery to induce OA *in vivo*, and intra-articular injection of erastin resulted in the increased ferroptosis of articular chondrocytes. Intra-articular injection of DFO (10 or 100 mg/kg) significantly reduced cartilage degeneration, as evaluated by HE and safranin O/fast green staining. In addition, we assessed the degree of OA through determining OARSI scores. Eight weeks after DMM surgery or the intra-articular injection of erastin, HE, and safranin O/fast green staining showed that the severity of cartilage damage in the DMM and erastin groups differed. Nevertheless, the DMM + DFO and erastin + DFO groups exhibited a smooth and complete cartilage surface, and the difference in OARSI scores was statistically significant (Figures 7A–C). In conclusion, the results of animal experiments suggested that DFO can slow the progression of OA in DMM mice.

## Discussion

As a common condition of the joints, the incidence of OA has increased in recent years. Previous findings have shown that various forms of chondrocyte death, inflammation, and oxidative stress affect the progression of OA. Ferroptosis is a unique non-apoptotic form of cell death that is characterized by the iron-dependent accumulation of lethal lipid ROS at overwhelming levels (Yang and Stockwell, 2016). The latest research by our group shows a specific relationship between chondrocyte ferroptosis and OA (Yao et al., 2021). Yan et al. found that DFO inhibited erastin-induced ferroptosis in primary cortical neurons (Zhang et al., 2020). However, it was not clear whether DFO can inhibit chondrocyte ferroptosis or alleviate the progression of OA. In our current study, we first confirmed that IL-1β can induce inflammation and destroy chondrocytes. In addition, it had positive effects on chondrocyte ferroptosis, such as its ability to inhibit SLC7A11 and GPX4; induce the excessive expression of P53, ACSl4, LOX15, and lpcat3; cause lipid ROS and ROS to accumulate; and increase MDA production in chondrocytes. These results indicated that ferroptosis occurred in chondrocytes induced by IL-1β. Moreover, IL-1β–induced OA-like changes in the chondrocytes could be suppressed by the ferroptosis inhibitor Fer-1, which means that the inhibition of ferroptosis in chondrocytes could alleviate chondrocyte inflammation and matrix destruction induced by IL-1β. Second, in erastin-treated chondrocytes, not only ferroptosis but also collagen II production decreased, MMP13 expression increased, and ROS accumulated, which further indicated that ferroptosis promoted OA-like changes in the chondrocytes, i.e., inflammation, oxidative stress, and matrix destruction.

In this study, DFO was shown to reverse the decrease in collagen II expression, attenuate the increase in MMP13 expression, and reduce ROS accumulation in chondrocytes *in vitro* and *in vivo*. In addition, the results of Western blotting indicated that DFO elevated GPX4 and SLC7A11 expression; inhibited the expression of ACSL4, LOX15, P53, and lpcat3; and decreased the accumulation of MDA and Fe^2+^, indicating that DFO may alleviate chondrocyte inflammation and matrix destruction induced by IL-1β through chondrocyte ferroptosis. For further verification, a model of chondrocyte ferroptosis was established through stimulation with erastin. Similarly, DFO increased chondrocyte GPx4 and SLC7A11 expression, which was inhibited by erastin; reduced erastin-induced P53, ACSL4, LOX15, and lpcat3 protein expression, which was promoted by erastin; and reduced intracellular MDA and Fe^2+^ accumulation, suggesting that DFO alleviates erastin-induced chondrocyte ferroptosis. In addition, DFO reversed the decrease in collagen II and mitigated the increase in MMP13 in erastin-treated chondrocytes. This evidence suggests that DFO protects chondrocytes from ferroptosis induced by erastin. Finally, DFO markedly improved mitochondrial structural disorder and functional damage in chondrocytes when ferroptosis was induced by treated with both IL-1β and erastin. In summary, our research has confirmed that chondrocyte ferroptosis is a significant factor that promotes the onset and development of OA, and DFO can protect chondrocytes and delay the progression of OA by inhibiting chondrocyte ferroptosis.

Nrf2 is not only the major antioxidant system in cells but also a critical protective factor that attenuates ferroptosis. The current study found that DFO activated the Nrf2 pathway and facilitated the transfer of Nrf2 and HO-1 into the nucleus when chondrocytes were treated with IL-1β. This finding suggests that DFO enhances the activation of Nrf2 to counter oxidative stress and protects chondrocytes from ferroptosis. In addition, knockdown of Nrf2 partially eliminated the DFO-induced increase collagen II levels and alleviating ferroptosis in chondrocytes induced by IL-1β. The results of Western blotting suggest that the Nrf2-mediated antioxidant system is vital for chondrocyte fate and that DFO may protect against IL-1β–induced chondrocyte ferroptosis through Nrf2.

To further prove the protective effect of DFO on OA *in vivo*, we established a C57BL/6 mouse model of OA by DMM surgery. Safranin O and HE staining showed that DFO could mitigate cartilage degeneration and significantly relieve the characteristics of OA, such as cartilage erosion and cartilage matrix loss. Consequently, DFO treatment significantly decreased the OARSI scores of the OA model mice.

Some limitations of this study are worth noting. First, we did not detect the protective effect of DFO on GPX4 activity, but DFO had a significant inhibitory effect on MDA, ACSL4, LOX15, and lipid ROS and improved mitochondrial damage. However, this limitation does not impair the conclusion that DFO inhibits the ferroptosis of chondrocytes. Second, unlike apoptosis, a gold standard method to detect ferroptosis has not yet been established. However, according to the definition of ferroptosis from the Nomenclature Committee on Cell Death, we validated the induction of chondrocyte ferroptosis by IL-1β or erastin, and preliminary evidence has shown that DFO may inhibit ferroptosis and delay OA progression *in vivo* and *in vitro*.

In summary, our results have confirmed the protective effect of DFO on OA *in vitro* and *in vivo*. Furthermore, we demonstrated that DFO attenuated inflammation and ECM degradation when chondrocytes were treated with IL-1β and erastin by mitigating chondrocyte ferroptosis. In addition, we found that DFO exerts its anti-ferroptotic and anti-inflammatory effects through the Nrf2 pathway. These findings suggest that DFO is a promising drug for the treatment of OA.

## Data Availability

The original contributions presented in the study are included in the article/supplementary material; further inquiries can be directed to the corresponding author.
