# Corrigendum: Deferoxamine alleviates osteoarthritis by inhibiting chondrocyte ferroptosis and activating the Nrf2 pathway

**DOI:** 10.3389/fphar.2023.1199951

**Published:** 2023-05-12

**Authors:** Zhou Guo, Jiamin Lin, Kai Sun, Jiayou Guo, Xudong Yao, Genchun Wang, Liangcai Hou, Jingting Xu, Jiachao Guo, Fengjing Guo

**Affiliations:** ^1^ Department of Orthopedics, Tongji Hospital, Tongji Medical College, Huazhong University of Science and Technology, Wuhan, China; ^2^ Michigan State University’s Broad College of Business, East Lansing, MI, United States; ^3^ Department of Orthopedic Surgery, The Second Affiliated Hospital of Chongqing Medical University, Chongqing, China; ^4^ Department of Pediatric Surgery, Tongji Hospital, Tongji Medical College, Huazhong University of Science and Technology, Wuhan, China

**Keywords:** osteoarthritis, chondrocytes, ferroptosis, deferoxamine, Nrf2

In the published article, there was an error in (Figure 3H) as published. (Due to the error of data filing, the fluorescence images of the Erastin group in Figure 3H was inserted incorrectly). The corrected (Figure 3H) and its caption (Erastin initiated inflammation responses and ECM degradation in chondrocytes that could be alleviated by Ferrostatin-1) appear below.

**FIGURE 3:**
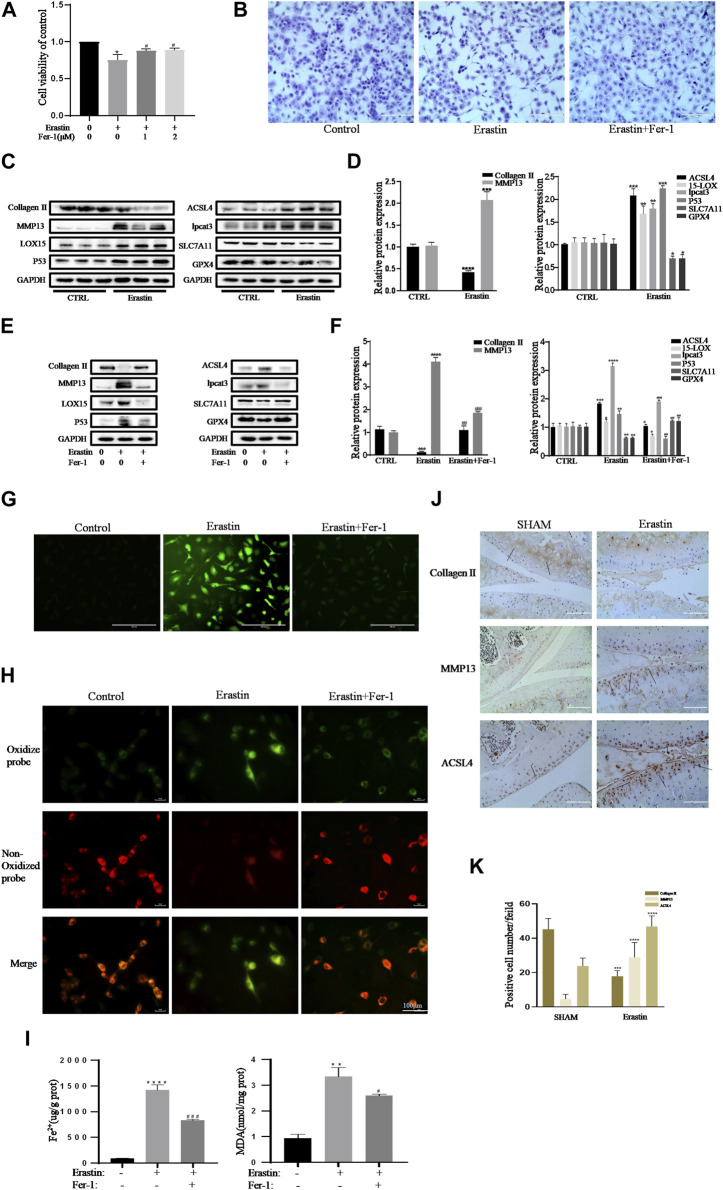
Erastin initiated inflammation responses and ECM degradation in chondrocytes that could be alleviated by Ferrostatin-1. **(A,B)** Cell viability determined by CCK-8 assay and toluidine blue staining. **(C,D)** The protein expression levels of collagen Ⅱ, MMP13, ACSL4, LOX15, lpcat3, P53, and SLC7A11 GPX4 when treated by erastin (5 μM) were detected by Western blot, and band density ratios of collagen Ⅱ, MMP13, ACSL4, LOX15, lpcat3, P53, and SLC7A11 GPX4 to GAPDH in the Western blots were quantified by densitometry (*n* = 3). **(E,F)** The protein expression levels of collagen Ⅱ, MMP13, ACSL4, LOX15, lpcat3, P53, and SLC7A11 GPX4 when treated by Erastin (5 μM) with fer-1 (1 μM) or equal volume of DMSO were detected by Western blot, and band density ratios of collagen Ⅱ, MMP13, ACSL4, LOX15, lpcat3, P53, and SLC7A11 GPX4 to GAPDH in the Western blots were quantified by densitometry (*n* = 3). **(G)** Intracellular ROS level detected by DCFH-DA fluorescent probe (scale bar: 200 µm). **(H)** Intracellular lipid-ROS level detected by C11 BODIPY fluorescent probe (scale bar: 100 µm). Red, reduced form of C11-BODIPY; green, oxidized form of C11-BODIPY. **(I)** The intracellular level of MDA and Fe^2+^ was determined using the MDA assay kit and iron assay kit (*n* = 3). **(J)** The collagen Ⅱ, MMP13, and ACSL4 expression levels in the cartilage samples were measured using immunohistochemistry staining. Dotted arrows indicate positive cells for MMP13 and ACSL4 and positive staining of collagen Ⅱ (scale bar: 100 µm). (K) Quantification of MMP13- and ACSL4-positive cells and collagen Ⅱ–positive staining in vivo. *p < 0.05 versus control or the sham group, **p < 0.01 versus control or the sham group, ***p < 0.001 versus control or the sham group, ****p < 0.0001 versus control or the sham group, #*p* < 0.05 versus IL-1β–treated group, ##*p* < 0.01 versus IL-1β–treated group, and ###*p* < 0.001 versus IL-1β–treated group. Error bars represent SD.

In the published article, there was an error in (Figure 5F) as published. (Due to the error of data filing, the fluorescence images of the Control group in Figure 5F was inserted incorrectly). The corrected (Figure 5F) and its caption (DFO alleviated chondrocytes ferroptosis and OA progress induced by erastin) appear below.

**FIGURE 5:**
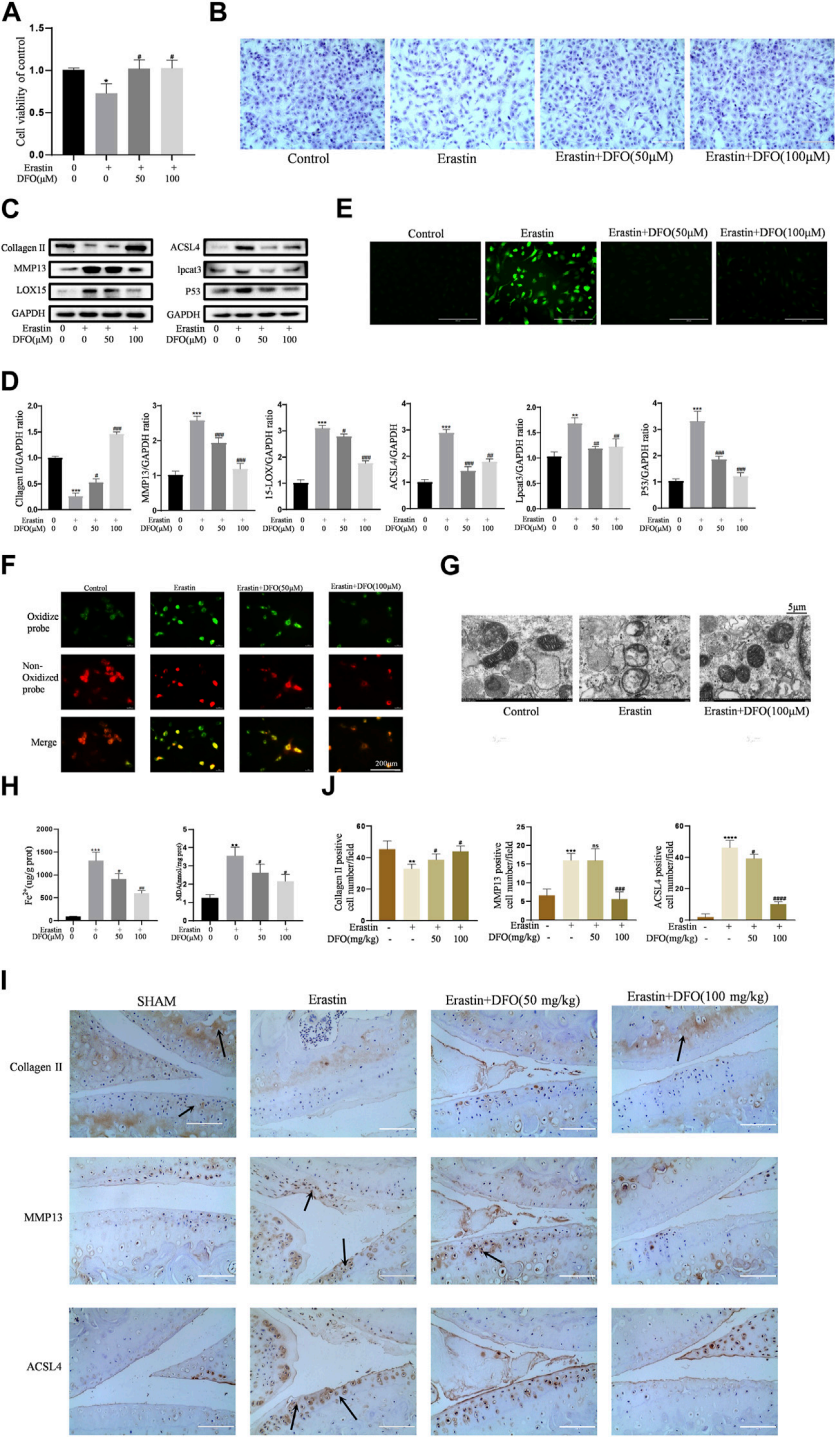
DFO alleviated chondrocytes ferroptosis and OA progress induced by erastin. **(A,B)** Cell viability determined by CCK-8 assay and toluidine blue staining. **(C,D)** The protein expression levels of collagen Ⅱ, MMP13, ACSL4, LOX15, lpcat3, and P53 when treated by Erastin (5 μM) with 50 and 100 μM DFO or equa volume of DMSO were detected by Western blot, and band density ratios of collagen Ⅱ, MMP13, ACSL4, LOX15, lpcat3, and P53 to GAPDH in the Western blots were quantified by densitometry (*n* = 3). **(E)** Intracellular ROS level detected by DCFH-DA fluorescent probe (scale bar: 200 µm). **(F)** Intracellular lipid-ROS level detected by C11 BODIPY fluorescent probe (scale bar: 200 µm). Red, reduced form of C11-BODIPY; green, oxidized form of C11-BODIPY. **(G)** The ultrastucture of mitochondria observed via transmission electron microscopy (scale bar: 5 µm). **(H)** The intracellular level of MDA and Fe2+ was determined using the MDA assay kit and iron assay kit *n* = 3). **(I)** The collagen Ⅱ, MMP13, and ACSL4 expression levels in the cartilage samples were measured using immunohistochemistry staining. Dotted arrows indicate positive cells for MMP13 and ACSL4 and positive staining of collagen Ⅱ (scale bar: 100 µm). **(J)** Quantification of MMP13- and ACSL4-positive cells and collagen Ⅱ–positive staining in vivo. *p < 0.05 versus control or the sham group, **p < 0.01 versus control or the sham group, ***p < 0.001 versus control or the sham group, ****p < 0.0001 versus control or the sham group, #*p* < 0.05 versus IL-1β–treated group or the DMM group, ##*p* < 0.01 versus IL-1β–treated group or the DMM group, ###*p* < 0.001 versus IL-1β–treated group or the DMM group, and ####*p* < 0.0001 versus IL-1β–treated group or the DMM group. Error bars represent SD.

In the published article, there was an error. (There is a clerical error in the Materials and Methods section).

A correction has been made to (**Materials and Methods**), *(Animal experiment)*. This sentence previously stated:

“(the sham group, DMM group, DMM + DFO (10 mg/kg) group, DMM + DFO (100 mg/kg) group, erastin group, erastin (15 mg/kg) + DFO (10 mg/kg) group, and erastin (15 mg/kg) + DFO (100 mg/kg) group.)”

The corrected sentence appears below:

“(the sham group, DMM group, DMM + DFO (10 mg/kg) group, DMM + DFO (100 mg/kg) group, erastin group, erastin (5 mg/kg) + DFO (10 mg/kg) group, and erastin (5 mg/kg) + DFO (100 mg/kg) group.)”

The authors apologize for these errors and state that this does not change the scientific conclusions of the article in any way. The original article has been updated.

